# Night sleep duration and risk of each lipid profile abnormality in a Chinese population: a prospective cohort study

**DOI:** 10.1186/s12944-020-01363-y

**Published:** 2020-08-15

**Authors:** Qiaofeng Song, Xiaoxue Liu, Wenhua Zhou, Shouling Wu, Xizhu Wang

**Affiliations:** 1 Department of Cardiology, Tangshan People’s Hospital, North China University of Science and Technology, No.65 Shengli Road, Lunan District, Tangshan, 063000 China; 2 Department of Cardiology, Kailuan Hospital, North China University of Science and Technology, Tangshan, 063000 China

**Keywords:** Sleep duration, Dyslipidemia, High-density lipoprotein cholesterol, Low-density lipoprotein cholesterol, Prospective cohort

## Abstract

**Background:**

To explore the associations between sleep duration and abnormalities in serum lipid levels in a Chinese population.

**Methods:**

A prospective study was conducted with 34,260 participants from the general Chinese population. Sleep duration was categorized as ≤5, 6, 7, 8 or ≥ 9 h. Each lipid profile abnormality was defined according to the Chinese Guidelines for the Prevention and Treatment of Dyslipidemia in Adults (2016). The Cox proportional hazards model was used to assess the association between sleep duration and dyslipidemia.

**Results:**

Compared with a 7 h sleep duration, long sleep duration (≥9 h) was significantly associated with low high-density lipoprotein cholesterol (HDL-C) levels (hazard ratio (HR): 1.24; 95% CI: 1.12–1.38). In subgroup analyses, the positive association between long sleep duration and low HDL-C level in men and in the different age groups was more pronounced than the association in women. No significant interactions were observed in the association between sleep duration and each abnormal serum lipid level by sex/age in the study population (P-interaction> 0.05).

**Conclusions:**

These findings suggest that long sleep duration is associated with low HDL-C level among the Kailuan community population.

## Introduction

Short or long sleep durations have been reported to be associated with a higher risk of diabetes mellitus [1–3], obesity [4, 5], hypertension [6, 7], cardiovascular disease (CVD) [8, 9] and atherosclerosis [10]. In fact, the mechanisms underlying these relationships are still unclear. In recent years, several studies have examined the links between sleep duration and risk factors for CVD, including lipids [11–21]. Dyslipidemia is characterized by high levels of total cholesterol (TC), low-density lipoprotein cholesterol (LDL-C), triglycerides (TG) and low levels of high-density lipoprotein cholesterol (HDL-C), and it increases the risk of CVD [22, 23] and hence remains an important issue in the field of health promotion and disease prevention. To prevent CVD, it is important to identify and improve the risk factors (including sleep duration) associated with serum TG, HDL-C or LDL-C levels.

Increasing evidence has suggested that short or long sleep durations might be associated with serum lipid profiles [11–21]. However, the findings have been inconsistent. Cross-sectional associations have been found between short sleep duration and lower HDL-C levels in adult American women with type 2 diabetes [19] and adult Japanese women from the general population [11]. A recent analysis of 2705 participants from the National Health and Nutrition Examination Survey showed that short sleep duration was associated with low HDL cholesterol [24]. In contrast, long sleep durations were found to be associated with low HDL-C levels among a Korean adult population from the Korean National Health and Nutrition Examination Survey [16]. A survey from the Coronary Artery Risk Development Study involving 503 black and white adults aged 32–51 years showed that long sleep duration was associated with increased future TC levels and TG levels but not with HDL-C [15]. However, a common limitation to these published studies is the lack of parameters related to snoring status and lack of a large sample cohort study. Little is known about the longitudinal relationships among factors. Based on this background, this study from Kailuan aimed to explore the association between sleep duration and each lipid profile abnormality stratified by age and sex.

## Methods

### Study design and participants

**Fig. 1:**
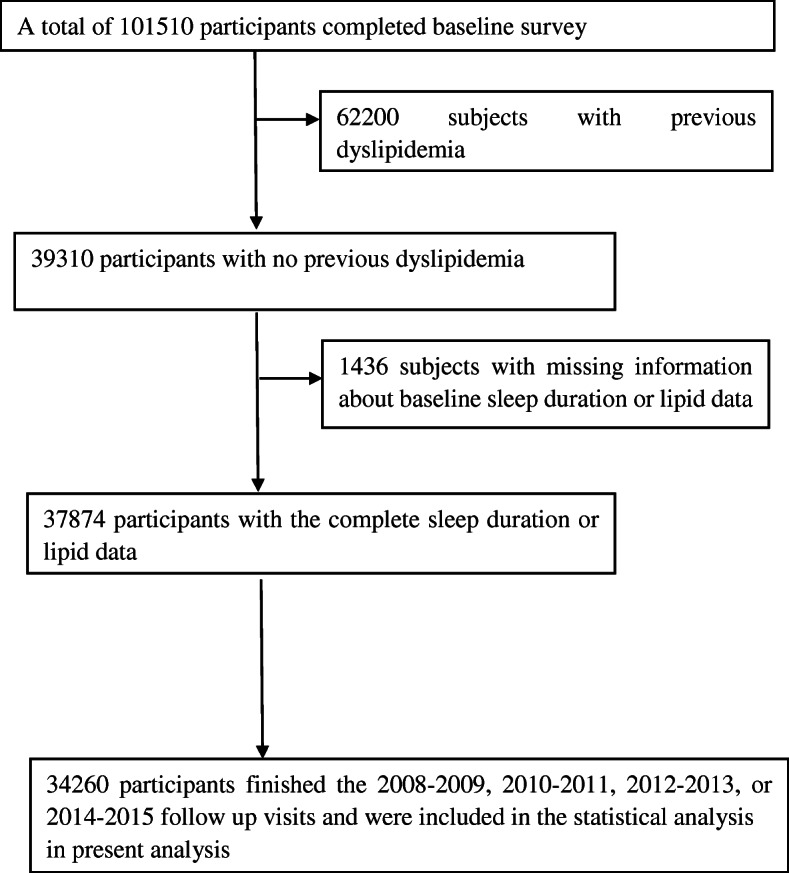
Flow chart of the present study

The Kailuan study was a prospective cohort study involving 101,510 participants (men: 81,110; women: 20,400; ages: 18–98 years) in the Kailuan community from June 2006 to October 2007 (visit 1) [25]. The participants were employees (including the retired) of Kailuan (Group) Co. Ltd. that represent all levels of the society, and they are employed as coalminers, administrators, secretaries, accountants, as well as the supportive and service staff, such as policemen, doctors, nurses, vendors, teachers. Of the 101,510 people who participated in the survey, 34,260 adults were included in the analysis after excluding the following participants: 62,200 subjects who had a history of dyslipidemia, 1436 subjects with missing information about baseline sleep duration or serum lipid profiles, and 3614 subjects without data for 2008–2009 (visit 1), 2010–2011 (visit 2), 2012–2013 (visit 3), and 2014–2015 (visit 4) follow-up visits (Fig. 1). The health interview survey was performed using self-administered structured questionnaires to obtain information on sociodemographic characteristics, health status, and health behaviors. Before the study, all doctors and nurses received rigorous unified training.

### Assessment of sleep duration

Sleep duration data were collected via self-reported answers to the question “How many hours of sleep have you had on an average at night in the preceding 3 months?” We divided sleep durations into five groups according to the responses: ≤5 h, 6 h, 7 h, 8 h, and ≥ 9 h. Additionally, participants were asked to answer “yes” or “no” to the question “Do you generally snore when you sleep?” [25, 26].

### Assessment of potential covariates

The clinical examination consisted of a medical history, a physical examination, anthropometric measurements, and self-administered questionnaires on lifestyle characteristics [25, 27], such as sleep duration,regular leisure-time physical activity, smoking habits, and dailyalcohol consumption. Physical activity was classified as ≥4 times per week and ≥ 20 min at a time (active physical activity), < 80 min per week, or none. Smoking status and drinking status were classified as never, former, or current according to the self-reported information. The questionnaire information was double-entered. The body mass index (BMI) was calculated as the weight (kg) divided by the height squared (meters^2^). Blood pressure was measured three times using a standardized sphygmomanometer while the participant was in a seated position. The average of the three measurements was used in this study.

Blood samples were drawn by trained phlebotomists from participants after an overnight fast for 8–12 h. Blood indicators, including TG, TC, LDL-C, HDL-C, fasting blood glucose (FBG), and high-sensitivity C-reactive protein (h-CRP) levels, were measured. TC and TG were measured enzymatically. HDL-C and LDL-C levels were measured using the direct test method [28]. All blood samples were analyzed using a Hitachi 747 autoanalyzer (Hitachi; Tokyo, Japan) [25]. All data was directly uploaded to the oracle database. Diabetes was defined as having a history of diabetes, the use of glucose-lowering agents, or an FBG ≥7 mmol/l. Hypertension was defined as a systolic blood pressure ≥ 140 mmHg, a diastolic blood pressure ≥ 90 mmHg, a history of hypertension and/or the use of antihypertensive agents.

### Diagnosis of each lipid profile abnormality

Each lipid profile abnormality was defined according to the Chinese guidelines on the prevention and treatment of dyslipidemia in adults (2016) [29]. Specifically, abnormal TC was defined as TC ≥5.2 mmol/L, abnormal TG were defined as TG ≥1.70 mmol/L, abnormal LDL-C was defined as LDL-C ≥ 3.4 mmol/L, and abnormal HDL-C was defined as HDL-C < 1.0 mmol/L.

### Statistical analyses

Continuous variables were described by their means ± standard deviations, and groups were compared using a one-way analysis of variance (ANOVA). Categorical variables were described as percentages and compared using the Chi-square test. The Cox proportional hazards model was used to estimate the HR for the incidence of each lipid profile abnormality in relation to sleep duration and baseline covariates. The follow-up time was calculated from the 2006 interview to the date when lipid profile abnormalities were detected, date of death, or date of the last attended interview in this analysis, whichever came first. For all models, the adequacy of the Cox proportional hazards model was assessed. Model 1 was adjusted for age and sex. Model 2 was further adjusted for the level of education, smoking, alcohol consumption, physical activity, and snoring. Model 3 was further adjusted for hypertension, diabetes mellitus, BMI, and h-CRP. Because transient changes in lipid profile (e.g., reversion from dyslipidemia to normal blood lipid) can impact the current results, the above analysis was repeated after excluding individuals with these conditions (Table 3, sensitivity analysis). To investigate whether age/sex acted as an effect modifier in the relationship between sleep duration and dyslipidemia, a regression model with an interaction term between age/sex and sleep duration was used. The statistical analysis was performed using SAS 9.4. All statistical tests were two-sided, and the significance level was set at 0.05.

## Results

The baseline characteristics of the study participants by sleep duration are shown in Table 1. The mean age of the participants was 49.13 years (range 20–97 years), and 76.26% were men. The percentages of participants who reported sleeping for ≤5 h, 6 h, 7 h, 8 h, and ≥ 9 h per night were 6.07, 15.72, 16.42, 60.05, and 1.74%, respectively. Age, sex, education, smoking, alcohol consumption, physical activity, hypertension, diabetes mellitus, and snoring symptoms differed between the sleep duration categories.

**Table 1:** Baseline characteristics according to sleep duration

Variable	Sleep duration (h)					*P* value
	≤5 (*n* = 2081)	6.0 (*n* = 5387)	7 (*n* = 5624)	8.0 (*n* = 20,573)	≥9 (*n* = 595)	
**Questionnaire-based data**						
Age, years	54.18 ± 12.29	51.38 ± 12.45	48.59 ± 12.89	48.27 ± 12.61	46.11 ± 14.91	< 0.001
Male, n (%)	1741(83.66)	4510(83.72)	4396(78.17)	15,052(73.16)	427(71.76)	< 0.001
High school or above, n (%)	386(18.55)	1337(24.82)	1941(34.51)	3656(17.77)	222(37.31)	< 0.001
Current smoker, n (%)	1148(55.17)	3016(55.99)	2949(52.44)	4883(23.73)	299(50.25)	< 0.001
Current alcohol consumption, n (%)	1127(54.16)	3167(58.79)	3183(56.60)	5097(24.78)	304(51.09)	< 0.001
Physically active, n (%)	556(26.72)	1216(22.57)	1186(21.09)	1647(8.01)	110(18.49)	< 0.001
Snoring, n (%)	458(22.01)	976(18.12)	891(15.84)	1260(6.12)	86(14.45)	< 0.001
**Exam-based data**						
Hypertension, n (%)	811(38.97)	1852(34.38)	1758(31.26)	7496(36.44)	163(27.39)	< 0.001
Systolic blood pressure, mmHg	128.70 ± 20.54	127.29 ± 20.04	125.14 ± 19.74	126.93 ± 20.16	123.01 ± 20.88	< 0.001
Diastolic blood pressure, mmHg	81.68 ± 11.23	81.13 ± 10.96	80.31 ± 10.97	81.85 ± 11.46	78.75 ± 11.42	< 0.001
Diabetes mellitus, n (%)	148(7.11)	315(5.85)	288(5.12)	1016(4.94)	38(6.39)	< 0.001
Fasting blood glucose, mmol/L	5.27 ± 1.38	5.21 ± 1.30	5.19 ± 1.20	5.22 ± 1.26	5.23 ± 1.42	0.915
Total cholesterol, mmol/L	4.37 ± 0.59	4.37 ± 0.57	4.35 ± 0.57	4.39 ± 0.57	4.29 ± 0.63	< 0.001
Triglycerides, mmol/L	0.98 ± 0.34	0.98 ± 0.33	0.97 ± 0.34	1.01 ± 0.33	0.95 ± 0.35	< 0.001
Low-density lipoprotein, mmol/L	2.14 ± 0.64	2.14 ± 0.64	2.15 ± 0.62	2.08 ± 0.68	2.01 ± 0.67	< 0.001
High-density lipoprotein, mmol/L	1.55 ± 0.36	1.55 ± 0.33	1.52 ± 0.32	1.55 ± 0.31	1.53 ± 0.32	< 0.001
Body mass index, kg/m^2^	24.11 ± 3.35	24.23 ± 3.32	24.25 ± 3.37	24.29 ± 3.37	24.04 ± 3.51	0.082
High-sensitivity C-reactive protein, mg/L	0.66(0.28–1.80)	0.64(0.25–1.70)	0.70(0.29–1.70)	0.65(0.22–1.87)	0.80(0.27–2.00)	0.088

Table 2 shows the characteristics of the study participants by dyslipidemia status. Participants with dyslipidemia were more likely to be men, have less than a high school education, be current smokers, be current drinkers, have a higher h-CRP level, have a higher blood pressure, have a higher prevalence of snoring symptoms, be overweight and have diabetes.

**Table 2:** Comparisons between patients with and without dyslipidemia in the Kailuan study

Variable No.	Dyslipidemia	Without dyslipidemia	*P* value
	*n* = 22,011	*n* = 12,249	
**Questionnaire-based data**			
Age, years	48.45 ± 12.18	50.36 ± 13.69	< 0.001
Male, n (%)	16,683(75.79)	9443(77.09)	< 0.05
Female, n (%)	5328(24.21)	2806(22.91)	
High school or above, n (%)	4982(22.63)	2560(20.90)	< 0.001
Current smoker, n (%)	8162(37.08)	4133(33.74)	< 0.001
Current alcohol consumption, n (%)	8606(39.10)	4272(34.88)	< 0.001
Physically active, n (%)	2989(13.58)	1726(14.09)	0.210
Snoring, n (%)	2479(11.26)	1192(9.73)	< 0.001
**Exam-based data**			
Hypertension, n (%)	7834(35.59)	4246(34.66)	0.08
Systolic blood pressure, mmHg	126.81 ± 19.81	126.58 ± 20.69	< 0.001
Diastolic blood pressure, mmHg	81.70 ± 11.23	80.92 ± 11.42	< 0.001
Diabetes mellitus, n (%)	1215(5.52)	590(4.82)	< 0.001
Fasting blood glucose, mmol/L	5.25 ± 1.27	5.156 ± 1.26	< 0.001
Total cholesterol, mmol/L	4.44 ± 0.56	4.26 ± 0.57	< 0.001
Triglycerides, mmol/L	1.04 ± 0.33	0.92 ± 0.32	< 0.001
Low-density lipoprotein, mmol/L	2.14 ± 0.67	2.03 ± 0.65	< 0.001
High-density lipoprotein, mmol/L	1.54 ± 0.32	1.57 ± 0.32	< 0.001
Body mass index, kg/m^2^	24.54 ± 3.35	23.74 ± 3.32	< 0.001
High-sensitivity C-reactive protein, mg/L	0.70(0.27–1.90)	0.58(0.20–1.58)	< 0.001

**Fig. 2:**
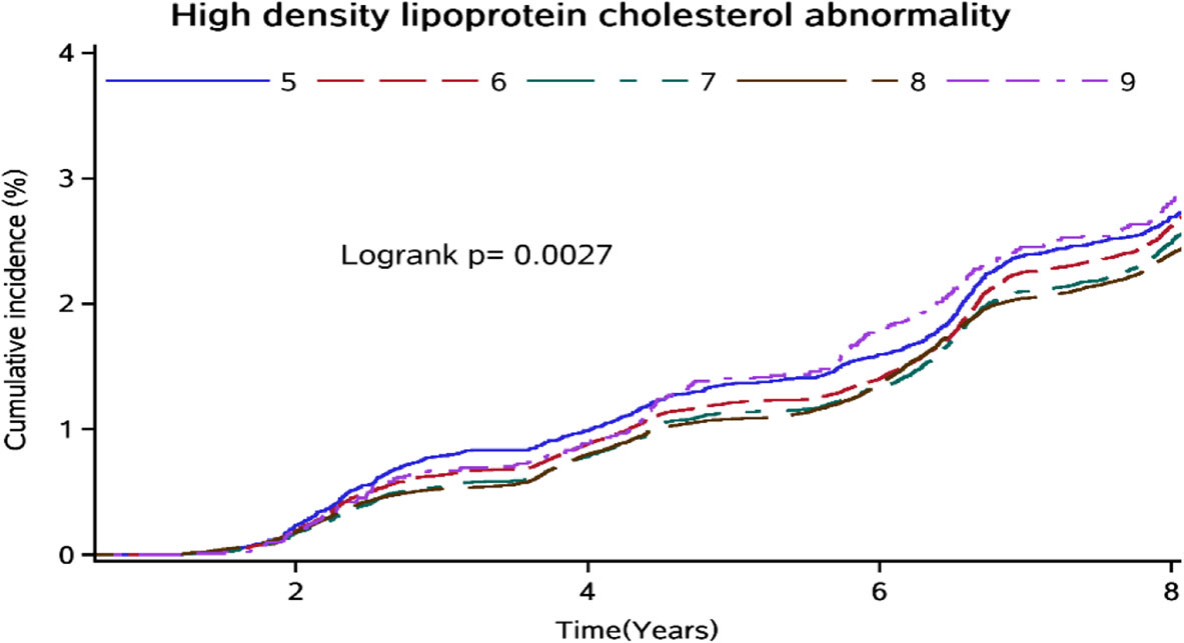
Cumulative incidence of high-density lipoprotein cholesterol abnormality among Kailuan Study participants in different sleep duration groups

The association between sleep duration and each abnormal serum lipid level was examined using multivariate Cox regression models. After adjusting for covariates, the long sleep duration (≥9 h) [16] was significantly associated with low HDL-C levels (HR: 1.24; 95% CI: 1.12–1.38) compared with a 7 h sleep duration (Table 3, Fig. 2). Moreover, the association between long sleep duration and the risk of low HDL-C levels remained significant even after excluding individuals with transient changes in lipid profile from the analysis (HR: 1.21; 95% CI: 1.07–1.35) (Table 3).

**Table 3:** Model results for the associations between sleep duration and each abnormal serum lipid level

Sleep duration (h)	Event, n(%)	Model 1	***P*** value	Model 2	***P*** value	Model 3	***P*** value	Sensitivity analysis^**a**^
		HR(95% CI)		HR(95% CI)		HR(95% CI)		HR(95% CI)
**TC**								
≤ 5	902(43.34)	0.95(0.88–1.02)	0.170	0.95(0.88–1.02)	0.174	0.96(0.89–1.04)	0.331	0.99(0.90–1.10)
6.0	2397(44.50)	1.00(0.94–1.06)	0.945	1.00(0.94–1.05)	0.893	1.00(0.95–1.06)	0.901	1.06(0.98–1.14)
7.0	2520(44.81)	reference		reference		reference		reference
8.0	8741(42.49)	0.93(0.89–0.97)	0.001	0.99(0.94–1.03)	0.527	0.99(0.94–1.04)	0.647	1.06(0.99–1.13)
≥ 9	237(39.83)	0.92(0.80–1.05)	0.216	0.92(0.81–1.05)	0.230	0.93(0.81–1.06)	0.278	0.98(0.83–1.16)
**TG**								
≤ 5	555(26.67)	0.97(0.88–1.06)	0.495	0.96(0.87–1.05)	0.373	0.99(0.90–1.09)	0.795	1.03(0.91–1.17)
6.0	1616(30.00)	1.04(0.97–1.11)	0.281	1.03(0.96–1.10)	0.413	1.03(0.97–1.11)	0.338	1.09(0.99–1.19)
7.0	1702(30.26)	reference		reference		reference		reference
8.0	6147(29.88)	1.01(0.96–1.06)	0.730	1.05(0.99–1.11)	0.094	1.04(0.98–1.10)	0.174	1.09(1.02–1.18)
≥ 9	188(31.60)	1.06(0.91–1.23)	0.445	1.07(0.92–1.24)	0.397	1.07(0.92–1.24)	0.406	1.08(0.89–1.31)
**LDL-C**								
≤ 5	405(19.46)	1.03(0.92–1.15)	0.643	1.02 (0.91–1.14)	0.768	1.04(0.93–1.17)	0.515	1.07(0.93–1.23)
6.0	1031(19.14)	1.03(0.94–1.12)	0.540	1.02(0.94–1.12)	0.584	1.03(0.94–1.12)	0.532	1.08(0.98–1.21)
7.0	1035(18.40)	reference		reference		reference		reference
8.0	4269(20.75)	1.18(1.10–1.26)	< 0.001	1.20(1.12–30)	< 0.001	1.21(1.12–1.30)	< 0.001	1.25(1.14–1.36)
≥ 9	100(16.81)	1.00(0.81–1.22)	0.969	0.99(0.81–1.22)	0.952	1.00(0.81–1.23)	0.985	0.88(0.67–1.14)
**HDL-C**								
≤ 5	1482(71.22)	1.01(0.95–1.07)	0.779	1.01(0.95–1.08)	0.655	1.02(0.96–1.08)	0.605	1.02(0.95–1.09)
6.0	3658(67.90)	1.01(0.97–1.06)	0.570	1.02(0.97–1.06)	0.505	1.01(0.97–1.06)	0.579	1.02(0.96–1.07)
7.0	3638(64.69)	reference		reference		reference		reference
8.0	13,610(66.15)	1.11(1.07–1.16)	< 0.001	1.11(1.07–1.15)	< 0.001	1.10(1.06–1.15)	< 0.001	1.11(1.06–1.16)
≥ 9	412(69.24)	1.27(1.15–1.41)	< 0.001	1.27(1.14–1.40)	< 0.001	1.24(1.12–1.38)	< 0.001	1.21(1.07–1.35)
**Dyslipidemia**								
≤ 5	1298(62.37)	0.96(0.90–1.02)	0.166	0.96(0.90–1.02)	0.955	0.98(0.92–1.04)	0.471	0.99(0.92–1.08)
6.0	3558(66.05)	1.04(0.99–1.10)	0.061	1.04(0.99–1.10)	0.061	1.05(1.00–1.10)	0.036	1.07(1.01–1.14)
7.0	3695(65.70)	reference		reference		reference		reference
8.0	13,081(63.58)	0.95(0.92–0.99)	0.012	1.01(0.97–1.05)	0.564	1.01(0.97–1.05)	0.509	1.08(1.02–1.14)
≥ 9	379(63.70)	0.98(0.88–1.09)	0.681	0.98(0.88–1.09)	0.706	0.98(0.88–1.09)	0.685	1.08(0.95–1.24)

Model 1 adjusted for age and sex

Model 2 was further adjusted for level of education, smoking, alcohol, physical activity, and snoring. Model 3 was further adjusted for hypertension, diabetes mellitus, body mass index, and high-sensitivity C-reactive protein

^a^ Adjusted for model 3 and further excluded individuals with transient changes in lipid profile (e.g., reversion from dyslipidemia to normal blood lipid)

Table 4 shows the association between sleep duration and each abnormal serum lipid level stratified by sex and age. Among men, there was a positive association between long sleep duration and low HDL-C levels (HR: 1.25; 95% CI: 1.12–1.41) in the fully adjusted model. Conversely, among women, there was a nonsignificant positive association between long sleep duration and low HDL-C levels in the fully adjusted model. A positive association between long sleep duration and low HDL-C levels was found in participants aged < 60 years (HR: 1.24; 95% CI: 1.10–1.39) but not participants aged ≥60 years (HR: 1.12; 95% CI: 0.90–1.40). However, no significant interactions were observed in the association between sleep duration and each abnormal serum lipid level by sex/age in the study population (P-interaction > 0.05).

**Table 4:** Model **3** results on the associations between sleep duration and abnormal serum lipid levels stratified by sex and age

Sleep duration (h)	Women		Men		Age < 60		Age ≥ 60	
	HR (95% CI)	***P*** value	HR (95% CI)	***P*** value	HR (95% CI)	***P*** value	HR (95% CI)	***P*** value
**TC**								
≤ 5	1.04(0.89–1.23)	0.596	0.95(0.85–1.06)	0.135	1.01(0.92–1.10)	0.868	0.88(0.75–1.05)	0.156
6.0	1.07(0.95–1.21)	0.269	1.01(0.99–1.12)	0.690	1.02(0.96–1.08)	0.530	0.95(0.83–1.09)	0.478
7.0	reference		reference		reference		reference	
8.0	0.98(0.89–1.07)	0.600	1.05(0.99–1.12)	0.737	0.99(0.94–1.04)	0.670	1.07(0.94–1.20)	0.307
≥ 9	1.04(0.82–1.33)	0.724	1.08(0.91–1.29)	0.270	0.97(0.84–1.12)	0.694	0.88(0.61–1.25)	0.464
**TG**								
≤ 5	1.11(0.89–1.40)	0.349	0.95(0.85–1.06)	0.338	1.04(0.94–1.16)	0.424	0.73(0.56–0.96)	0.022
6.0	1.14(0.96–1.34)	0.134	1.01(0.94–1.09)	0.735	1.04(0.97–1.12)	0.297	0.99(0.81–1.21)	0.941
7.0	reference		reference		reference		reference	
8.0	1.08(0.95–1.23)	0.224	1.05(0.99–1.12)	0.105	1.05(0.99–1.12)	0.090	1.00(0.84–1.21)	0.920
≥ 9	1.21(0.88–1.67)	0.238	1.08(0.91–1.29)	0.388	1.08(0.92–1.27)	0.339	1.11(0.70–1.76)	0.664
**LDL-C**								
≤ 5	1.30(1.01–1.67)	0.039	0.98(0.86–1.11)	0.710	1.17(1.03–1.33)	0.019	0.77(0.60–1.00)	0.047
6.0	1.09(0.90–1.34)	0.380	1.01(0.92–1.11)	0.856	1.06(0.96–1.16)	0.257	0.93(0.76–1.14)	0.492
7.0	reference		reference		reference		reference	
8.0	1.33(1.15–1.55)	< 0.001	1.17(1.08–1.27)	< 0.001	1.21(1.12–1.31)	< 0.001	1.25(1.05–1.48)	0.013
≥ 9	1.26(0.86–1.84)	0.232	0.94(0.73–1.20)	0.600	1.05(0.84–1.31)	0.668	0.81(0.48–1.37)	0.427
**HDL-C**								
≤ 5	0.99(0.85–1.16)	0.921	1.02(0.96–1.09)	0.511	1.01(0.94–1.09)	0.782	1.03(0.92–1.15)	0.624
6.0	1.03(0.92–1.16)	0.633	1.01(0.96–1.06)	0.630	1.02(0.97–1.08)	0.396	0.97(0.88–1.07)	0.588
7.0	reference		reference		reference		reference	
8.0	1.06(0.97–1.16)	0.178	1.10(1.06–1.15)	< 0.001	1.10(1.05–1.15)	< 0.001	1.11(1.02–1.21)	< 0.05
≥ 9	1.14(0.91–1.44)	0.249	1.25(1.12–1.41)	< 0.001	1.24(1.10–1.39)	< 0.001	1.12(0.90–1.40)	0.317
**Dyslipidemia**								
≤ 5	1.14(0.98–1.31)	0.086	0.94(0.88–1.01)	0.098	1.03(0.96–1.11)	0.418	0.85(0.74–0.98)	0.027
6.0	1.15(1.01–1.28)	0.010	1.03(0.98–1.09)	0.243	1.07(1.02–1.12)	0.011	0.99(0.89–1.11)	0.902
7.0	reference		reference		reference		reference	
8.0	1.03(0.95–1.12)	0.452	1.03(0.98–1.07)	0.245	1.01(0.97–1.05)	0.700	1.10(0.99–1.22)	0.062
≥ 9	1.13(0.92–1.39)	0.253	0.97(0.85–1.10)	0.587	1.02(0.91–1.14)	0.763	0.89(0.67–1.18)	0.427

Model 3 was adjusted for age (stratified by sex), sex (stratified by age), education, smoking, alcohol, physical activity, snoring, hypertension, diabetes mellitus, body mass index, and C-reactive protein

## Discussion

This prospective study demonstrated that long sleep duration was associated with an increased risk of future low HDL-C level. This association was observed independent of age, sex, BMI, h-CRP, smoking habits, alcohol consumption, physical activity, snoring, education, diabetes mellitus, and hypertension. However, no associations were revealed between sleep duration and the risk of future high TG, TC, or LDL-C levels. A sensitivity analyses further confirmed these findings.

Several studies have reported the associations between serum lipid profiles and sleep duration, although the results of these studies have been inconsistent [11–13, 18, 20, 30]. A National Health and Nutrition Survey in Japan showed a U-shaped (≤5 h, ≥9 h) relationship between sleep duration and a high level of TG and between sleep duration and a low level of HDL-C [11]. Consistent with this finding, a study from the China Health and Nutrition Survey (2009) involving 8574 adults showed that both shorter (≤6 h) and longer (≥10 h) sleep durations were associated with higher risks of abnormal serum lipid profiles [18]. However, two cross-sectional studies conducted in the USA and Japan showed that self-reported sleep duration was not associated with hypercholesterolemia [20, 30]. On the other hand, the Kansai Healthcare Study in Japan reported that moderate (5–7 h) and/or long (≥7 h) sleep durations decreased the risk of future low HDL-C and high TG levels [12]. Additionally, Juliana C. Chan et al. concluded that long (> 9.25 h) sleep duration was associated with lower risks of high TC and LDL-C in children and adolescents [13]. These prospective findings suggest that long sleep duration is associated with low HDL-C levels among the Chinese population. Differences in the age distribution of the study population, lifestyle, socioeconomic status, incomplete control for confounding factors, or different categorization and cutoff points of sleep duration may explain the inconclusive associations thus far.

Several studies have found sex differences in the associations between sleep duration and abnormal serum lipid levels [17, 18]. Results from the National Health Interview Survey 2008 found sex differences in the association between sleep duration and hypercholesterolemia, with a positive association found between sleep duration ≤5 h and hypercholesterolemia in women and an inverse association found between sleep duration ≥8 h and hypercholesterolemia in men [17]. Data from the China Health and Nutrition Survey (2009) showed that both short and long sleep durations were associated with higher risks of abnormal serum lipid profiles in women but not in men [18]. However, the present study found no significant interaction between sleep duration and sex with respect to abnormal serum lipid levels and showed that differences occurred in the sleep duration pattern between men and women. However, it is unclear whether these differences induce sex differences in the association between sleep duration and abnormal serum lipid levels. Additionally, the current study found no significant interaction between sleep duration and age with respect to abnormal serum lipid levels. Further research is needed to investigate the association between sleep duration and serum lipid levels.

Moreover, the biological mechanism responsible for the association between long sleep duration and a low HDL-C level is not easily explained. First, several previous studies have shown that prolonged sleep duration may be associated with obesity [31], hypertension [6], diabetes and glucose intolerance [32, 33]. Second, sleep duration and abnormal lipids often share some common risk factors, such as smoking [34], alcohol consumption [35], and lower socioeconomic status [36]. Third, inflammation is one of the most important biological pathways because long sleep durations can lead to increased levels of inflammatory markers. This study also found that the level of h-CRP was higher in participants with dyslipidemia than in those without dyslipidemia (Table 2).

### Study strengths and limitations

The major strengths of the current study include its large sample size and the availability of information on potential confounders and mediators. However, this study has some limitations. First, sleep duration data were collected through self-reported questionnaires. The use of more precise measures of sleep-related variables, such as polysomnographies, may reduce variability; however, these techniques are logistically unfeasible to perform in large epidemiological studies such as ours. Second, data on sleep quality, such as difficulty falling asleep, excessive daytime sleepiness, and obstructive sleep apnea (OSA), were not collected in the current study. A very recent study has shown that the association between OSA and impaired lipid profiles is partly determined genetically [37]. However, snoring status was adjusted as an alternative confounder since patients with OSA often have symptoms of snoring status. Third, some time-varying variables, such as lipid-lowering therapy, physical activity, smoking habits, daily alcohol consumption, onset of systemic diseases during the period, and statins medication use at each follow-up, may modify serum lipids levels or sleep duration and have a favorable effect on the current results. For example, statins have a well-known side effect of tiredness and fatigue, which may result in long sleep times [38, 39]. Paradoxically, patients who take statins may have better lipid profiles but long sleep times. Fourth, the proportion of individuals in the “9 or more hours of sleep” category is very small compared with the proportions in other categories. Thus, the estimates in this group might be unstable and influenced by employment status. Fifth, as participants were recruited from Tangshan (an industrial city located in northern China), the cohort is not nationally representative, and the generalizability of the current results to other geographic regions and to people of other racial/ethnic groups is not known. Finally, the current study was an observational study and we investigated only the association between baseline sleep duration and risk of dyslipidemia; thus, we did not consider changes at each time point in sleep duration. Indeed, any subsequent alterations in sleep duration may lead to a nondifferential misclassification and potential underestimation of the sleep–dyslipidemia association.

## Conclusions

In conclusion, the results of this study indicate that long sleep duration is associated with a low HDL–C level among the Kailuan community population. The amount of sleep might play a key role in the risk of future lipid profile abnormalities. Encouraging and supporting individuals to pursue 7 h of sleep per night may have significant beneficial effects towards stemming the growing incidence of dyslipidemia in China.

## Data Availability

The datasets used and/or analyzed during the current study are not available.

## Abbreviations

LDL-C: Low-density lipoprotein cholesterol
HDL-C: High-density lipoprotein cholesterol
CVD: Cardiovascular disease
TC: Total cholesterol
TG: Triglyceride
BMI: Body mass index
FBG: Fasting blood glucose
h-CRP: High-sensitivity C-reactive protein
HR: Hazard ratio
